# Brown Seaweed Byproduct Extracts Improve Intestinal Motility and Auto-Inflammation in Mice with Loperamide-Induced Constipation

**DOI:** 10.3390/foods13132037

**Published:** 2024-06-27

**Authors:** Eun-Jeong Koh, Kwang-Soon Shin, In Yung Sunwoo, Junseong Kim, Woon-Yong Choi

**Affiliations:** 1 Jeju Bio Research Center, Korea Institute of Ocean Science and Technology (KIOST), Jeju 63349, Republic of Korea; kej763@kiost.ac.kr (E.-J.K.); iysunwoo@kiost.ac.kr (I.Y.S.); junseong@kiost.ac.kr (J.K.); 2 Department of Food Science and Biotechnology, Kyonggi University, Suwon 16227, Republic of Korea; ksshin@kyonggi.ac.kr; 3 Department of Marine Technology & Convergence Engineering (Marine Biotechnology), KIOST School, University of Science and Technology (UST), Daejeon 34113, Republic of Korea

**Keywords:** *Sargassum fusiforme*, *Sargassum fulvellum*, seaweed byproducts, constipation, intestinal motility, inflammation

## Abstract

*Sargassum fusiforme* and *Sargassum fulvellum* are types of brown algae used for their nutritional value and medicinal properties, including anti-inflammatory, antioxidant, and anticancer effects. Despite their importance in various industries, many seaweed byproducts containing dietary fiber and polysaccharides are discarded in landfills. These byproducts can be recycled and repurposed for different applications. In this study, we investigated the impact of *S. fusiforme* food processing byproducts (MbP-SFF) and *S. fulvellum* food processing byproducts (MbP-SFV) on improving intestinal motility and reducing inflammation in mice with constipation induced by loperamide. To evaluate this, mice were orally administered 500 mg/kg/day of the byproducts once daily for 8 days. Constipation was induced by 5 mg/kg/day of loperamide for two days after oral administration for 6 days. Each sample contained approximately 70% carbohydrates. MbP-SFF had 52.0% mannuronic acid and 18.8% guluronic acid, while MbP-SFV had 36.9% mannuronic acid and 32.9% guluronic acid. These byproducts enhanced fecal excretion and intestinal motility by modulating inflammatory responses. Furthermore, they restored the balance of the gut microbiota disrupted by loperamide, increasing beneficial *Bifidobacterium* and reducing harmful *Staphylococcus aureus*. Overall, MbP-SFF and MbP-SFV improved intestinal motility and inflammation by influencing the gut microbiota and inflammatory responses in a loperamide-induced mouse model. These byproducts show potential as ingredients in functional foods aimed at enhancing gut health, potentially reducing waste disposal costs and addressing environmental concerns associated with their utilization.

## 1. Introduction

*Sargassum fusiforme* and *Sargassum fulvellum* are an edible seaweed found along the coasts of Korea, Japan, and China. These seaweeds are commonly used as functional foods and medicine due to their richness in proteins, polysaccharides, and dietary fibers, which offer antiaging, anti-inflammatory, and gut health benefits [1,2]. Historically, *Sargassum* species have been integral to traditional diets and medicine in East Asia. These seaweeds have been used for centuries to treat various ailments, highlighting their potential as therapeutic agents [3].

In recent times, extracts of these seaweeds have been used in hangover drinks in Korea for their proven hangover-relieving effects [4]. The manufacturing process of these hangover drinks generates over 5000 kg of byproducts from the seaweed extracts, which are then discarded. The disposal of these marine byproducts is costly and leads to environmental pollution.

Brown seaweed has traditionally been known for its effectiveness in relieving constipation, and this has been reaffirmed through several studies [5]. In addition, it has been reported that *Sargassum fusiforme* and *S. fulvellum* improve intestinal motility in loperamide-induced constipation mice [6,7].

Constipation is a gastrointestinal condition that results in discomfort, including poor appetite, difficulty in passing stool, dry and lumpy stool, pain during bowel movements, abdominal pain, and distension [8]. Various factors, including diet, colonic propulsion, lifestyle, and drug side effects, contribute to constipation [9,10]. Multiple mechanisms are involved in the pathogenesis of constipation, including the dysfunction of intestinal motility, imbalance in gut microbiota, and nerve-related disorders [11,12].

Numerous studies have demonstrated a relationship between the gut microbiota balance and constipation. Alterations in the composition of the gut microbiota of patients with constipation have been reported. Specifically, the abundance of pathogenic bacteria, including *Campylobacter jejuni* and *Pseudomonas*, is increased in such patients, with a concomitant decrease in beneficial bacteria, such as *Bifidobacterium* and *Bacteroides* spp. [13,14,15]. These changes in the gut microflora can affect various aspects of intestinal function, including absorption, intestinal motility, and the nervous system, ultimately leading to constipation [16].

Probiotics improve constipation by regulating gut motility, the immune system, and inflammation [17]. Butyrate-producing bacteria promote the formation of a mucosal layer, leading to the attenuation of constipation symptoms [18]. Wang et al. demonstrated that *Bifidobacterium* reduces the levels of inflammatory factors, including interleukin 1β (IL-1β), nuclear factor kappa-B (NF-κB), and tumor necrosis factor alpha (TNF-α), thereby relieving the symptoms of constipation [19]. Additionally, lactobacillus metabolites were reported by Cervantes-Barragan to regulate TH17 cell/Treg balance and intestinal homeostasis, thereby attenuating gut inflammation [20]. However, the mechanisms underlying the interactions between obligate bacteria and intestinal motility, immune responses, and inflammation during constipation are not fully understood.

Although previous studies have reported the positive effects of these two seaweeds on gut health [1,7], the effects of extracts of their byproducts on constipation-induced inflammation have not been thoroughly examined. In this study, we evaluated the effects of these extracts on intestinal motility and inflammation in mice treated with loperamide, representing an animal model of constipation. Additionally, we investigated the potential utilization of extracts of *S. fusiforme* (MbP-SFF) and *S. fulvellum* (MbP-SFV) byproducts as commercial products to reduce the cost and environmental issues associated with their disposal.

## 2. Materials and Methods

### 2.1. Preparation of Marine Byproduct Extracts

Marine brown algae (*S. fusiforme,* MbP-SFF and *S. fulvellum*, MbP-SFV) were provided by BKBio (Jeju, Republic of Korea). The byproducts were extracted using a five-step extraction method. First, water was added at a ratio of 30 times the number of byproducts, and the mixture was extracted at 95 °C for 20 h. The resulting extracts were then concentrated using a vacuum evaporator (EYELA, Gyeonggi, Republic of Korea). The concentrate of these seaweeds was precipitated using 95% ethanol at a ratio of three times the amount of concentrate for 24 h. After precipitation, pectin was separated from the precipitate using a mesh filter (TISCH Scientific, Cleves, OH, USA). The pectin was then dried at 50 °C for 24 h.

### 2.2. Analysis of the Composition of Marine Byproduct Extracts

The composition of marine byproduct extracts was analyzed at the Institute of Agricultural Science, Chungnam National University, Daejeon, Republic of Korea. The analyses adhered to the protocols outlined by the American Organization of Analytical Chemists (AOAC) for the determination of crude protein (AOAC 2000.11), crude lipid (AOAC 991.36), and crude fiber (AOAC 962.09). The total carbohydrate content was quantified by deducting the cumulative crude protein, lipid, and ash content from the overall composition [21].

### 2.3. Analysis of Sugar Components in Marine Byproduct Extracts

The sugar composition of algal polysaccharides was analyzed using a 3-methyl-1-phenyl-2-pyrazoline-5-one (PMP)-derivative assay [22,23]. Briefly, the polysaccharide was hydrolyzed by treatment with 2 M trifluoroacetic acid at 121 °C for 90 min, and the hydrolyzed monosaccharides were treated with PMP under basic conditions at 70 °C for 60 min. The reactant was neutralized using hydrochloric acid and subsequently separated using a chloroform/H_2_O two-phase solvent system; only the H_2_O layer (PMP-monosaccharide fraction) was collected. The PMP–monosaccharide fractions were analyzed using a high-performance liquid chromatograph (LC-20AD, Shimadzu Ltd., Kyoto, Japan) equipped with a UV/vis detector (SPD-20A, Shimadzu Ltd.) and a C18 column (Acclaim TM 120, Thermo Fisher, Waltham, MA, USA).

### 2.4. Animals and Experimental Design

Seven-week-old male ICR mice were purchased from Hana Biotech (Gyeonggi, Republic of Korea) and were maintained at the Southeast Medi-Chem Institute (Busan, Republic of Korea). Mice were housed for 1 week under a 12 h light/dark cycle at 22 ± 2 °C under 50 ± 10% relative humidity. This study was approved by the Institutional Animal Care and Use Committee of Southeast Medi-Chem Institute (IACUC approval number SEMI-21-009).

The mice (*n* = 7 for each group) were randomly divided into five groups. They were orally administered 5 mg/kg/day loperamide (C), 120 mg/kg/day Psyllium husk powder (P), 500 mg/kg/day MbP-SFF or MbP-SFV, or an equal volume of vehicle saline solution (N) once daily for eight days. Constipation was induced via a gavage of 5 mg/kg/day loperamide for two days after oral administration for 6 days. After the induction of constipation, bowel improvement was evaluated. We followed all institutional and national guidelines regarding the care and use of laboratory animals.

### 2.5. Fecal Weight and Water Content

Fecal weight and water content were analyzed for 14 days. We collected feces samples after the induction of constipation for 5 h. The samples were weighed and dried thoroughly in an oven to obtain their dry weight. Fecal weight and water content were calculated as follows:

Fecal weight (g) = Wet weight − dry weight

$$\mathrm{Fecal}\;\mathrm{water}\;\mathrm{content}\;\left(\%\right)=\frac{\mathrm{Wet}\;\mathrm{weight}\;-\;\mathrm{dry}\;\mathrm{weight}}{\mathrm{Wet}\;\mathrm{weight}}\times 100$$

### 2.6. Intestinal Transit Rate

The intestinal transit rate was measured on day 15. The mice were gavaged with 5 mg/kg/day loperamide for 30 min after treatment with P, MbP-SFF, MbP-SFV, or saline solution and subsequently administered 0.5% phenol red (in 1.5% methyl cellulose). After 20 min, the mice were euthanized using CO_2_ asphyxiation and cervical dislocation. The entire intestine was removed from the sacrificed mice and its length was measured.

The intestinal transit rate was calculated as follows:

$$\mathrm{Intestinal}\;\mathrm{transit}\;\mathrm{rate}\;\left(\%\right)=\frac{\mathrm{Distance}\;\mathrm{of}\;\mathrm{transported}\;\mathrm{phenol}\;\mathrm{red}}{\mathrm{Total}\;\mathrm{length}\;\mathrm{of}\;\mathrm{the}\;\mathrm{small}\;\mathrm{intestine}}\times 100$$

### 2.7. Biochemical Analysis

Intestinal tissue was homogenized in phosphate-buffered saline (PBS) and centrifuged at 12,000× *g* for 20 min at 4 °C to obtain the supernatant, which was used to determine the levels of inflammatory factors.

The levels of NF-κB, TNF-α, and prostaglandin E2 in the intestinal tissue were measured using enzyme-linked immunosorbent assay (ELISA) kits (CUSABIO, Houston, TX, USA), as per the manufacturer’s instructions. The kits used for the assay employ the double-antibody sandwich method to determine the levels of the inflammatory factors in mouse tissues. The absorbance was measured at 450 nm using a microplate reader (Molecular Devices, San Jose, CA, USA).

Mouse blood was collected by cardiac puncture and placed aseptically in blood collection tubes. To collect serum, blood was coagulated for 1 h at room temperature and then centrifuged at 13,000× *g* for 15 min at 4 °C. The levels of immunoglobulin A (IgA) and immunoglobulin G (IgG) in blood serum were measured using an ELISA kit (Abcam Technology, Cambridge, UK), according to the manufacturer’s instructions.

### 2.8. Assessment of Gut Microbiota Growth

Fecal samples were collected and immediately stored at −20 °C to preserve bacterial integrity. For bacterial quantification, 0.1 g of each sample was aseptically transferred into a sterile tube containing 9 mL of physiological saline. The sample was then shaken for 30 min to ensure the proper dispersion of bacteria. Serial dilutions of the suspensions were prepared and plated on selective agar media suitable for the growth of *Staphylococcus aureus* and *Bifidobacterium*, such as BS agar (BD Biosciences, Franklin Lakes, NJ, USA). The agar plates were incubated at 37 °C for 48–72 h under appropriate anaerobic or microaerophilic conditions to facilitate the growth of the target bacteria. Following incubation, the number of colonies of *S. aureus* and *Bifidobacterium* on the plates was counted, and the results were expressed as log10 (colony forming units [CFUs]) per gram of fecal content.

### 2.9. Statistical Analysis

All statistical analyses were performed using the Statistical Program for StatView (ver.5.0.1) (SAS Institute Inc., Cary, NC, USA). Group comparisons were performed using one-way analysis of variance (ANOVA). One-way ANOVA with Fisher’s PLSD post hoc test was used to analyze the differences between the mean values of the groups. Data are presented as the mean ± standard deviation (SD). A *p*-value < 0.05 was considered to indicate statistical significance.

## 3. Results

### 3.1. Composition of Marine Byproduct Extracts

We measured the major nutrients and dietary fiber content of the marine byproduct extracts remaining after the manufacturing of hangover drinks to confirm their potential as functional ingredients. As shown in Table 1, the marine byproduct extract had 72–73% carbohydrate, 3–5% crude protein, 0.2–0.4% crude fat, 0.1–0.2% crude fiber, 22–23% crude ash, and 64–67% dietary fiber.

**Figure 1:**
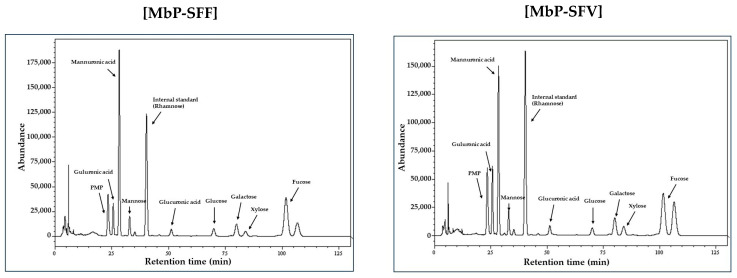
Sugar component analysis of marine byproduct extracts. HPLC chromatogram of eight polysaccharides from MbP-SFF and MbP-SFV.

Additionally, we employed a PMP-derivative assay to analyze the algal polysaccharides and confirm the sugar composition. The chromatogram in Figure 1 shows peaks for eight polysaccharides isolated from MbP-SFF and MbP-SFV. The molar ratio of mannuronic acid was the highest in MbP-SFF, and it constituted 52.0% of the total polysaccharides, followed by 18.8% guluronic acid and 14.9% fucose. Similarly, MbP-SFV contained 36.9% mannuronic acid, 32.9% guluronic acid, and 14.4% fucose (Table 2). These findings suggest that the high content of polysaccharides, including mannuronic acid, guluronic acid, and fucose, in MbP-SFF and MbP-SFV may contribute to the improvement in constipation and the gut environment.

### 3.2. Effects of Marine Byproduct Extracts on Fecal Excretion and the Small Intestinal Transit

**Figure 2:**
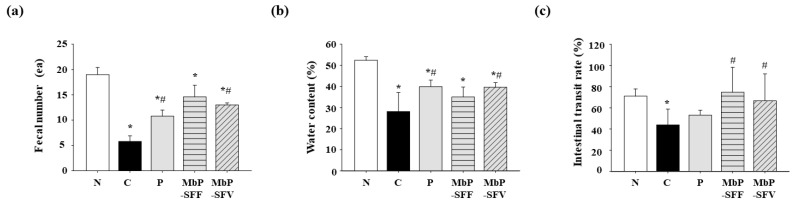
Effects of marine byproduct extracts on fecal excretion and the small intestinal transit. (**a**) Feces were collected after inducing constipation for 5 h and then the samples were weighed. Weight was calculated according to the formula mentioned in the text. (**b**) Feces were dried in an oven for measuring dry sample weight. Water content was calculated using the formula mentioned in the text. (**c**) Mice were administered 5 mg/kg/day loperamide for 30 min after P, SF, S, or saline treatment, and then the intestines were stained with phenol red solution. Intestinal rate was calculated according to the formula mentioned in the text. The experiments were conducted in septuplicate. The data are indicated as the mean ± SD. * *p* < 0.05, compared with the vehicle group. # *p* < 0.05, compared with the control group. Abbreviations: N, vehicle; C, control, 5 mg/kg/day loperamide; P, positive control, 120 mg/kg/day Psyllium husk; MbP-SFF, 500 mg/kg/day extract of *Sargassum fusiforme* byproducts; MbP-SFV, 500 mg/kg/day extract of *Sargassum fulvellum* byproducts.

We analyzed fecal excretion, including the number of fecal samples and the water content, to evaluate the effects of the extracts on bowel movements in mice with constipation. *Psyllium husk* powder was used as a positive control approved by the Korean Ministry of Food and Drug Safety as a “functional ingredient for enhancing bowel movement”. As shown in Figure 2a, the fecal number in the control group (C, 5.8 ± 1.1 ea) was significantly decreased compared with that in the vehicle group (N, 19.0 ± 1.4 ea) (*p* < 0.05). However, the amount of feces was increased in the MbP-SFV (13.0 ± 0.4 ea) and positive control (P, 10.8 ± 1.2 ea) groups (*p* < 0.05).

Moreover, the water content was reduced by loperamide (C, 28.1 ± 9.0%) (*p* < 0.05) (Figure 2b). MbP-SFV (39.6 ± 2.2%) and the positive control (P, 39.9 ± 3.1%) significantly elevated the water content compared with that in the control group (C, 28.1 ± 9.0%) (*p* < 0.05). These results indicated the successful establishment of a constipation model using loperamide and the effects of marine byproduct extracts in improving bowel movement.

Additionally, we analyzed the small intestinal transit rate to elucidate whether the marine byproduct extracts affected the intestinal motility, leading to improved bowel movement. As shown in Figure 2c, loperamide clearly reduced the intestinal transit rate (C, 43.9 ± 14.9%) compared with that in the vehicle group (N, 71.0 ± 6.9%) (*p* > 0.05). Conversely, the administration of marine byproduct extracts significantly increased the intestinal rate (MbP-SFF, 74.7 ± 23.5%; MbP-SFV, 66.7 ± 25.3%) (*p* > 0.05). These data indicated that MbP-SFF and MbP-SFV ameliorated the effects on bowel movement and intestinal motility in mice with loperamide-induced constipation.

### 3.3. Effects of Marine Byproduct Extracts on the Levels of Inflammatory Factors and Immune Response

**Figure 3:**
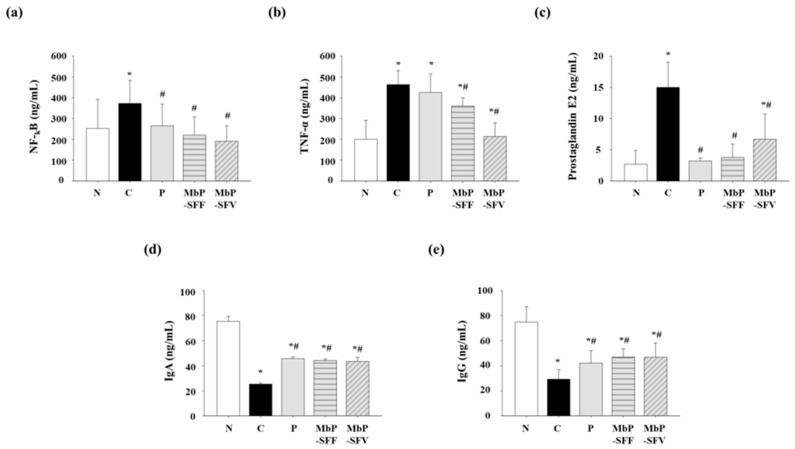
Effects of marine byproduct extracts on the levels of inflammatory factors and prostaglandin E2. (**a**–**e**) The protein levels of inflammatory factors (NF-κB, TNF-α, and prostaglandin E2) and immunoglobulins (IgA and IgG) in the colon were analyzed using ELISA. The experiments were conducted in septuplicate. The data are indicated as the mean ± SD. * *p* < 0.05, compared with the vehicle group. # *p* < 0.05, compared with the control group. Abbreviations: N, vehicle; C, control, 5 mg/kg/day loperamide; P, positive control, 120 mg/kg/day Psyllium husk; MbP-SFF, 500 mg/kg/day extract of *Sargassum fusiforme* byproducts; MbP-SFV, 500 mg/kg/day extract of *Sargassum fulvellum* byproducts.

We examined the levels of inflammatory factors (NF-κB and TNF-α) and prostaglandin E2 to evaluate the effects of marine byproduct extracts on inflammation in constipated mice (Figure 3a–c). The levels of NF-κB were increased in the constipation group (C, 372.4 ± 111.4 pg/mL) compared with that in the vehicle group (N, 253.3 ± 138.3 pg/mL) (*p* > 0.05). Administration of MbP-SFF and MbP-SFV decreased the loperamide-induced levels of NF-κB (MbP-SFF, 220.2 ± 87.6 pg/mL; MbP-SFV, 190.8 ± 74.3 pg/mL) and TNF-α (MbP-SFF, 360.3 ± 39.5 pg/mL; MbP-SFV, 214.2 ± 65.6 pg/mL) (*p* > 0.05). Additionally, the levels of prostaglandin E2 (C, 15.0 ± 4.0 pg/mL) were increased by loperamide compared with that in the vehicle group (N, 2.7 ± 2.2), and the trend was similar as that for NF-κB and TNF-α (*p* > 0.05). Extracts of marine byproducts also reduced the levels of prostaglandin E2 (MbP-SFF, 3.8 ± 2.1 pg/mL; MbP-SFV, 6.7 ± 4.0 pg/mL) (*p* > 0.05). These results indicate that marine byproduct extracts modulate proinflammatory factors in mice with loperamide-induced constipation.

In addition, we investigated whether constipation affected the gut immune system and whether marine byproduct extracts could regulate the gut immune system in constipation by measuring IgA and IgG levels (Figure 3d,e). The IgA levels were decreased in the loperamide-treated group (C, 25.33 ± 1.05 ng/mL) compared with those in the vehicle group (N, 75.37 ± 4.08 ng/mL) (*p* > 0.05). Administration of MbP-SFF and MbP-SFV ameliorated the reduction in IgA levels caused by constipation. Furthermore, the constipation group (C, 25.33 ± 1.05 ng/mL) exhibited reduced IgG levels (*p* > 0.05). In contrast, IgG levels were increased by the marine byproduct extracts (MbP-SFF, 46.94 ± 6.53 ng/mL; MbP-SFV, 46.80 ± 11.34 ng/mL) (*p* > 0.05). These findings indicate that constipation affects the gut immune response and that marine byproduct extracts can modulate these immune responses.

### 3.4. Effects of Marine Byproduct Extracts on the Intestinal Microbiota

**Figure 4:**
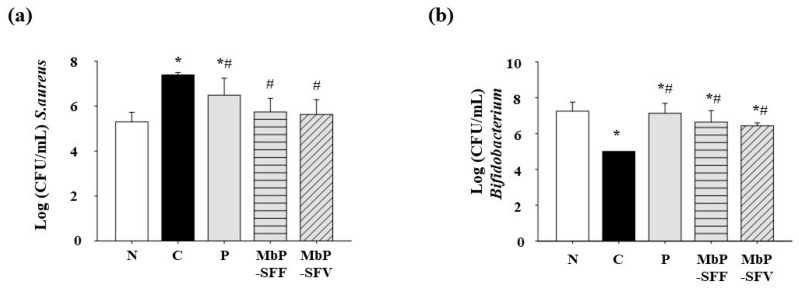
Effects of marine byproduct extracts on the intestinal microbiota. (**a**,**b**) The number of intestinal bacteria (*S. aureus* and *Bifidobacterium*) in the feces was analyzed using the spread plate method. The number of colonies of *S. aureus* and *Bifidobacterium* on the plates was counted, and the results are expressed as log10 (colony forming units [CFUs]) per gram of fecal content. All experiments were conducted in duplicate. The data are indicated as the mean ± SD. * *p* < 0.05, compared with the vehicle group. # *p* < 0.05, compared with the control group. Abbreviations: N, vehicle; C, control, 5 mg/kg/day loperamide; P, positive control, 120 mg/kg/day Psyllium husk; MbP-SFF, 500 mg/kg/day extract of *Sargassum fusiforme* byproducts; MbP-SFV, 500 mg/kg/day extract of *Sargassum fulvellum* byproducts.

We analyzed the abundance of pathogenic and beneficial bacteria (*S. aureus* and *Bifidobacterium*, respectively) to determine the effect of marine byproduct extracts on the alteration of intestinal microbiota by constipation. The abundance of *S. aureus* was markedly increased in constipated mice (C, 7.39 ± 0.10 CFUs/mL) compared with that in the vehicle group (N, 5.30 ± 0.43 CFUs/mL). Administration of MbP-SFF (5.74 ± 0.61 CFUs/mL) and MbP-SFV (5.63 ± 0.66 CFUs/mL) reduced the loperamide-induced increase in the abundance of *S. aureus* (*p* > 0.05) (Figure 4a). Conversely, the loperamide-treated group (C, 5.0 ± 0.0 CFUs/mL) showed a significant decrease in the abundance of *Bifidobacterium* compared with that in the vehicle group (N, 7.25 ± 0.5 CFUs/mL). MbP-SFF (6.64 ± 0.64 CFUs/mL) and MbP-SFV (6.43 ± 0.17 CFUs/mL) increased the abundance of *Bifidobacterium* (*p* > 0.05) (Figure 4b). These results indicate that the marine byproduct extracts affect the intestinal microbiota, which is altered by constipation.

## 4. Discussion

To determine the therapeutic and nutraceutical potential of marine byproduct extracts in gastrointestinal motility disorders and to elucidate their mechanisms of action, we used a mouse model of loperamide-induced constipation. Loperamide is commonly used to alleviate diarrheal symptoms by suppressing smooth muscle tone and peristalsis. It can also be used to enhance fecal continence by increasing the anal sphincter tone [24]. However, loperamide can cause adverse effects on intestinal motility, including constipation, bloating, and abdominal pain. Considering these adverse effects, it has been used to establish a constipated animal model for in vivo studies of gastrointestinal diseases [25]. We administered the samples for 7 days before treating the mice with loperamide to induce constipation. The mice treated only with loperamide showed suppressed water secretion, delayed stool evacuation, and prolonged intestinal luminal transit (Figure 2). These results indicated the successful establishment of the mouse model of constipation.

Edible brown seaweeds grown in Korea, Japan, and China are recognized for their high nutritional content [1,26]. They contain dietary fiber, polysaccharides, and bioactive compounds, such as alginic acid, fucoidan, and fucoxanthin, which exhibit biomedical effects on gastrointestinal diseases, oxidative stress, and inflammation [26,27]. In view of the elucidated biological activities of these ingredients, they have been utilized for the development of functional materials, including functional foods, medicines, and cosmetic products. Recently, a Korean company developed a hangover drink using these two seaweeds, resulting in the generation of byproducts that increase disposal costs and environmental pollution associated with the use of these seaweeds [4]. Considering these issues, we investigated the potential use of these two byproducts for the development of functional materials.

First, we measured the major nutrient and dietary fiber content of MbP-SFF and MbP-SFV. These extracts contained carbohydrates and dietary fiber (Table 1). Carbohydrates are composed of various polysaccharides that exert beneficial effects, including protection of the gut epithelial barrier, anti-inflammatory properties, and modulation of the immune system through mechanisms that can be dependent on or independent of the microbiota [28]. Considering the abundance of carbohydrates, we analyzed the content of eight polysaccharides, Including mannuronic acid, guluronic acid, and fucose. Mannuronic acid, guluronic acid, and fucose were abundant in MbP-SFF and MbP-SFV (Table 2). Notably, mannuronic acid and guluronic acid are components of alginate. Alginates are a type of dietary fiber found in brown seaweeds and are composed of linear chains of mannuronic and guluronic acids [29]. Alginate polysaccharides have various biological activities, including antioxidant and anti-inflammation effects [30]. These reports suggest that MbP-SFF and MbP-SFV may improve gut health and exert anti-inflammatory effects, likely due to their alginate polysaccharides.

To test our hypothesis, we measured intestinal motility, including fecal excretion and the level of inflammatory factors. MbP-SFV improved the bowel movement and intestinal motility (Figure 2). The other extract significantly increased the intestinal transit rate, indicating a tendency toward enhanced movement within the intestines rather than an elevation in fecal extraction.

Constipation is related to inflammatory factors. Choi et al. reported that loperamide-induced constipation stimulated PI3K/AKT signaling pathways and the expression of inflammatory factors, such as NF-κB, TNF-α, and IL-1α [31]. We measured the level of inflammatory factors to elucidate the relationship between constipation and inflammation, as well as the effects of marine byproduct extracts. Administration of loperamide led to an increase in NF-κB, TNF-α, and prostaglandin E2 levels (Figure 3). However, MbP-SFF and MbP-SFV suppressed the inflammation induced by constipation.

Impairment of the intestinal microbiota is another factor that leads to constipation. Generally, the abundance of pathogenic bacteria increases, whereas that of *Bifidobacterium* and *Bacteroides* spp. decreases [13,15]. Additionally, changes in the gut microbiota can lead to an increase in proinflammatory bacteria or a decrease in beneficial bacteria, contributing to local inflammation in the intestines [17]. We hypothesized that the altered motility patterns caused by loperamide could disrupt the balance of the gut microbiota, leading to proinflammatory effects. We also investigated whether these two seaweed extracts could improve gut health. To support our hypothesis, we analyzed the abundance of *S. aureus* and *Bifidobacterium*. Constipation induced by loperamide changed the gut flora in a pattern similar to that observed in the studies mentioned above, and extracts of the two marine byproducts ameliorated this imbalance in the microbiota.

*Bifidobacterium* produces short-chain fatty acids (SCFAs), which elevate fecal humidity and stimulate intestinal peristalsis, thereby relieving constipation symptoms, such as hard stools and evacuation disorders [32,33]. In addition, SCFAs produced by obligate bacteria regulate the microbiota, intestinal immune response, and inflammation, thereby sustaining the intestinal environment. Activation of G protein-coupled receptors by SCFAs promotes IgA production and suppresses NF-κB activity, thereby modulating immune responses and inflammation in the intestine [34].

Recent studies have demonstrated a relationship between constipation and SCFAs. SCFA levels decrease in constipated subjects and mice [35,36]. Specifically, clinical trials and in vivo studies have reported an increase in the production of inflammatory factors, such as TNF-α and NF-κB, in the constipation group [31,37]. We hypothesize that a reduction in *Bifidobacterium* and SCFAs due to constipation contributes to inflammation. However, the mechanism of action of SCFAs is not fully understood, warranting further studies to confirm this hypothesis. In the future, we intend to investigate the association between SCFAs and gut microbiota, as well as the effects of marine byproducts on the gut environment in constipated mice.

In conclusion, we have demonstrated that MbP-SFF and MbP-SFV improve intestinal motility in mice with loperamide-induced constipation. Moreover, these brown seaweed byproduct extracts reduced gut inflammation by suppressing inflammatory factors, including NF-κB and TNF-α. Additionally, these extracts ameliorate the imbalance in the gut environment caused by constipation by modulating pathogenic and beneficial bacteria, specifically *S. aureus* and *Bifidobacterium*. These results suggest that SCFAs may be produced by *Bifidobacterium*, leading to the inhibition of inflammatory factors, such as NF-κB, TNF-α, and prostaglandin E2. However, this hypothesis could not be fully validated in the present study. Further research should be conducted to elucidate the mechanism by which an increase in SCFAs generated by *Bifidobacterium* contributes to the suppression of gut inflammation and improvement in intestinal motility in constipation.

## Figures and Tables

**Table 1:** Composition of MbP-SFF and MbP-SFV. The contents of carbohydrate, crude protein, fat, fiber, ash, and dietary fiber in MbP-SFF and MbP-SFV are expressed as percentages. Values are expressed as mean ± standard deviation (n = 3).

Nutritional Composition (%)	MbP-SFF	MbP-SFV
Carbohydrate	72.45 ± 0.8	71.57 ± 1.1
Crude protein	3.36 ± 0.1	4.44 ± 0.4
Crude fat	0.82 ± 0.7	0.98 ± 1.0
Crude fiber	0.40 ± 0.4	0.17 ± 0.1
Crude ash	22.69 ± 0.2	22.53 ± 0.3
Dietary fiber	66.68 ± 1.2	63.02 ± 1.8

**Table 2:** The molar ratio of sugar composition in MbP-SFV and MbP-SFF. Sugar composition of MbP-SFF and MbP-SFV is expressed as mole percentages. Values are expressed as mean ± standard deviation (n = 3).

Sugar Component (mole %)	MbP-SFF	MbP-SFV
Guluronic acid	18.8 ± 0.1	32.9 ± 0.3
Mannuronic acid	52.0 ± 0.7	36.9 ± 0.7
Mannose	2.8 ± 0.1	2.8 ± 0.0
Glucuronic acid	4.3 ± 0.0	4.6 ± 0.2
Glucose	2.1 ± 0.0	1.7 ± 0.0
Galactose	3.4 ± 0.1	4.1 ± 0.0
Xylose	1.6 ± 0.1	2.6 ± 0.0
Fucose	14.9 ± 0.8	14.4 ± 0.5

## Data Availability

The original contributions presented in the study are included in the article, further inquiries can be directed to the corresponding author.
